# Transport properties of electron small polarons in a V_2_O_5_ cathode of Li-ion batteries: a computational study†

**DOI:** 10.1039/c9ra02923k

**Published:** 2019-06-21

**Authors:** Panuwat Watthaisong, Sirichok Jungthawan, Pussana Hirunsit, Suwit Suthirakun

**Affiliations:** School of Chemistry, Institute of Science, Suranaree University of Technology Nakhon Ratchasima Thailand 30000 suthirak@sut.ac.th +66-44-224-886; Center of Excellence in Advanced Functional Materials, Suranaree University of Technology Nakhon Ratchasima Thailand 30000; School of Physics, Institute of Science, Suranaree University of Technology Nakhon Ratchasima Thailand 30000; Thailand Center of Excellence in Physics (ThEP), Ministry of Higher Education, Science, Research and Innovation (MHESI) 328 Si Ayutthaya Road Bangkok Thailand 10400; National Nanotechnology Center (NANOTEC), National Science and Technology Development Agency (NSTDA) 111 Thailand Science Park Pathum Thani Thailand 12120

## Abstract

Employing the first-principles plane-wave approach, we explored the behavior of electron transport in the V_2_O_5_ cathode. Polaron migrations along different crystallographic directions in the presence and absence of Li^+^ ions were systematically examined using linear interpolation (LE) and nudged elastic band (NEB) methods. We find that the NEB calculations, based on structural optimizations of TS structures, generally exhibit lower hopping barriers than those obtained from the LE calculations. Both methods consistently predict that the [010] hopping, in the presence and absence of a nearby Li^+^ ion, is kinetically least favorable since the migration involves displacements of rigid 3-coordinated O atoms. Computations based on the LE method reveal anisotropic polaron mobilities where the estimated hopping frequencies within the layer are approximately one order of magnitude higher than the normal. The prediction based on the LE calculations is consistent with the experimental results. Lithiation dramatically affects the behavior of polaron movement. It significantly increases the reaction energies and hopping barriers due to the strong polaron-ion interaction. In addition, it is predicted that polaron hopping in the V_2_O_5_ cathode is non-adiabatic where lithiation has negligible effects on the adiabaticity.

## Abstract 2

Mechanisms and properties of the electron transport at the V_2_O_5_ cathode of Li-ion batteries were studied by means of first-principles computations.

## Graphical abstract

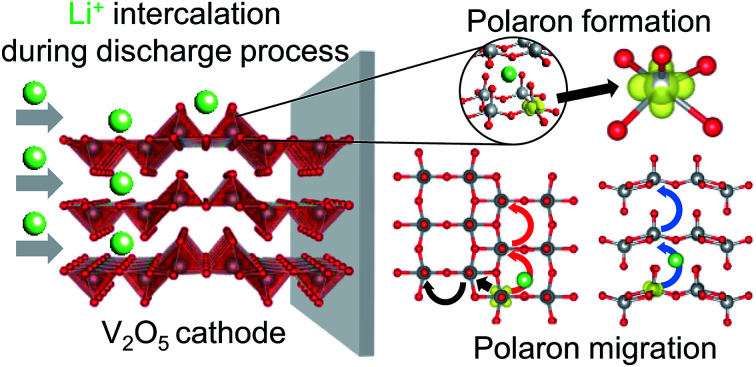

## 1 Introduction

Currently, Li-ion batteries (LIBs) have been successfully commercialized and become an irreplaceable energy storage device for new energy vehicles and various portable electronic devices because of their high-energy density and long lifetime.^1–4^ The performance of the LIBs are determined by the specific capacity of electrodes and the potential difference between the cathode and the anode.^5^ Recently, α-V_2_O_5_ has emerged as a promising cathode material due to its unique physical and chemical properties.^6–9^ Its layered structure is responsible for the high energy density since it provides accommodation for small ions such as Li^+^ and Na^+^, and other multivalent cations such as Mg^2+^, Ca^2+^, and Y^3+^.^10–14^ Despite its high Li capacity, bottlenecks in the diffusion of ions and electrons lead to slow electrochemical kinetics and poor cyclability.^15–17^ Therefore, it is of importance to understand the behavior of charge transport at the V_2_O_5_ cathode.

At the beginning of the discharge process, Li^+^ ions diffuse from the anode and start to intercalate at the electrolyte–cathode interface. At the same time, electrons swiftly travel from the anode *via* an external circuit to the cathode to maintain charge neutrality.^4,18–20^ The added electrons at the cathode thereby reduce transition metal centers (in this study V^5+^ → V^4+^ of V_2_O_5_) where electron “small polarons” are created.^3,4,21–24^ The small polarons at the reduced V centers induce local distortion of the surrounding lattice. These polarons, then, undergo thermally activated hopping through the lattice and finally meet their Li-ion counterparts. Once the separated charges (polarons and ions) reconcile, the Coulomb attraction between electron polarons and Li^+^ ions enforces close proximity of ion-polaron pairs. It has been suggested that the Li^+^ ion and its associated polaron may diffuse together in a coupled fashion, as studied in several cathode materials including Li_*x*_V_2_O_5_,^25–27^ Li_*x*_FePO_4_,^28^ Li in TiO_2_,^29,30^ and Li_2_FeSiO_4_.^31^

As the discharge progresses, lithiation at the cathode induces phase transformation of Li_*x*_V_2_O_5_ as *x* increases; α (0 < *x* < 0.33) → ε (0.33 < *x* < 0.80) → δ (0.88 < *x* < 1.00) → γ (1.0 < *x* < 2.0).^32–37^ The geometrical and electronic structures among Li_*x*_V_2_O_5_ phases are different leading to variation of the charge transport mechanisms in the materials.^27,38^ As illustrated in a computational study, the calculated polaron migration barriers are varied in different Li_*x*_V_2_O_5_ phases as α-Li_0.083_V_2_O_5_ (∼0.34 eV), ε-Li_0.083_V_2_O_5_ (∼0.37 eV), and ζ-Li_0.083_V_2_O_5_ (∼0.24 eV).^39^ In addition, the phase transformation upon lithiation can bring about dislocations at the interface between the parent lattice and the Li-rich phase. Such creation of phase boundaries has been found to greatly diminish Li-ion diffusivity at the V_2_O_5_ cathode.^40^

Several strategies have been suggested to boost the electrochemical kinetics of the V_2_O_5_ cathode. For instance, to avoid the slow diffusion kinetics at the interface, one could operate the cathode in the voltage range where the material maintains its original phase.^39^ Meta-stable V_2_O_5_ polymorphs have also been reported to substantially reduce migration barriers of Na- and Mg-ion diffusion.^39,41,42^ Decreasing the particle size and making novel microstructures of V_2_O_5_ can shorten the diffusion path length and lead to improved ion diffusivity.^27^ In addition, various metal elements such as Cr,^43^ Cu,^44,45^ Al,^46^ and Sn^47,48^ have been used as doping atoms to improve the electrochemical performance of the V_2_O_5_ cathode.

Intercalation of Li inevitably generates excess electrons which undergo concomitant reduction of V ions in the V_2_O_5_ lattice. Behavior of the injected electrons plays a role in phase transformation and charge transport kinetics of the V_2_O_5_ cathode. As evidence in some experimental studies that the charge transport kinetics of oxide-cathodes (V_2_O_5_ and LiFePO_4_) are limited by electron transfer rather than the cation diffusion itself.^49,50^ It is the objective of this current study to better understand the behavior of electron transport at its most fundamental mechanism. We examine from first-principles the electronic structures and transport properties of electron polarons at early discharge stage at the V_2_O_5_ cathode of LIBs. While studies based on the use of conventional density functional theory (DFT) with generalized gradient approximation (GGA) were successful at determining crystal structure and cell potentials of Li-intercalated V_2_O_5_,^51,52^ recent studies used a variant of Kohn–Sham method, introducing an on-site Hubbard model correction (DFT+U)^53^ to properly describe the localization behavior of excess electrons in the V_2_O_5_ lattice.^25,26,39,54–57^ Therefore, to describe the formation of a small polaron and its transport properties, we invoked the *U* correction scheme to account for proper electron localizations and correct, at least in part, the self-interaction error inherent in local or semi-local exchange-correlation approximations. In particular, we calculated energy barriers of various electron migration paths in the presence and absence of a Li^+^ ion to further explain the characteristic of electronic conduction in the V_2_O_5_ cathode.

## 2 Computational details

All calculations were carried out using the spin-polarized DFT+U approach with periodic supercell model as implemented in the Vienna Ab Initio Simulation Package (VASP 5.3).^58–60^ The exchange and correlation functional was approximated using the optimized vdW-DF functional (modified versions of the vdW-DF of Dion *et al.*)^61^ as implemented in VASP to describe weak attractions between V_2_O_5_ layers; where its original GGA exchange functional has been replaced by an optimized Perdew–Burke–Ernzerhof (PBE) functional.^62^ The projector augmented-wave (PAW) scheme^63,64^ was used to describe the nuclei and core electronic states (V 1s2s2p, O 1s, and Li 1s) where their valence-electron wave functions were expanded in plane-wave basis with a cutoff energy of 400 eV for electronic structure calculations and geometry optimizations. In the self-consistent field (SCF) procedure, we used a Gaussian smearing technique with a smearing width of 0.05 eV. The final energies were obtained by extrapolating to zero smearing width. The convergent tolerance of SCF was set to 1 × 10^−6^ eV. For the DFT+U variant,^53^ the *U* − *J* value of 4.0 eV was chosen for V 3d electrons, as previously used by Scanlon *et al.*^54^ to properly describe the behavior of extra electrons in reduced V_2_O_5_. The *U* value was validated to match the positions of the mid-gap state found in UPS/XPS spectra^65^ for the oxygen-vacancy system and Li-intercalated system. It was reported that the positions of the mid-gap state for both systems are equivalent suggesting that the used *U* value can be apply in the presence and absence of Li in the V_2_O_5_ system.

The V_2_O_5_ unit cell was optimized using a 5 × 11 × 11 Monkhorst–Pack k-mesh^66^ and a kinetic energy cutoff of 700 eV to obtain the equilibrium lattice parameters and ion positions. Such a high energy cutoff is needed to avoid an underestimation of the equilibrium volume caused by the Pulay stress. The optimized unit cell was then used to construct a 1 × 3 × 3 supercell of V_36_O_90_ to further used for examining the behavior of electron transport in the presence and absence of Li^+^ ions. These calculations were carried out using a 2 × 2 × 2 Monkhorst–Pack k-mesh. All geometry optimizations were ceased when the calculated residual forces were lower than 0.02 eV Å^−1^. Electronic structure calculations of projected density of states (PDOS) were carried out using the tetrahedron smearing method with Bloch corrections. The gamma-centered *k*-point of 2 × 2 × 2 was found to be sufficient to obtain a convergence of the calculated DOS. Using a denser 4 × 4 × 4 k-mesh yields a negligible difference of the PDOS results.

Formation of a small polaron in the pure V_2_O_5_ system was done by adding an extra electron into the lattice. Charge neutrality of the supercell is ensured by a compensating homogeneous background charge. Initial perturbation of oxygen atoms around a vanadium atom was carried out to break the lattice symmetry which allowed localization of the added electron at the V center. On the other hand, for the Li-intercalated system, no electrons were added to the charge density since the excess charge carrier was provided by the ionized Li^+^ ion.

We examined various possible paths of polaron migration to study transport properties of electrons in the cathode material. We adopted two approaches to determine the migration paths and calculate the hopping barriers. (i) The linear interpolation (LE) approach is used to approximate the migration path between two equilibrium structures obtained from two independent optimizations of internal coordinates. Single-point energy calculations of all other configurations except for the two equilibrium structures were carried out to yield the polaron hopping barrier. (ii) We employed the nudged elastic band (NEB) method implemented in VTST tools^67^ to determine the minimum energy path of polaron transfer. The NEB approach allows for ionic relaxation of each configuration along the migration path. We reported a deviation between the two methods. The calculated barriers obtained from these methods are considered adiabatic which imply the validity of the Born–Oppenheimer approximation.

The adiabaticity of a polaron transfer can be determined if we know the adiabatic barrier and the electronic coupling constant (*V*_AB_). We employed the method described in the computational work by Adelstein and co-workers to estimate the *V*_AB_.^68^*V*_AB_ is a half of the energy difference between the adiabatic bonding and antibonding electronic states at the transition state (TS) configuration, *V*_AB_ = ½Δ*E*_12_. In practice, we can estimate the Δ*E*_12_ from the positions of the two gap states in the calculated density of states of the TS. The bonding state (below the Fermi energy) and the antibonding state (above the Fermi energy) must be in the gap between the valence band and the conduction band and should be linear combinations of the initial and final polaron states.

## 3 Results and discussion

### 3.1 Atomic and electronic structures

Vanadium pentoxide (α-V_2_O_5_) develops an orthorhombic crystal structure in *Pmmn* space group with the lattice parameters obtained from experiments: *a* = 11.51 Å, *b* = 3.56 Å, and *c* = 4.37 Å.^69^ As depicted in Fig. S1,† its primitive cell contains two formula units, four V atoms, and ten O atoms, in series of layers stacked along the *z*-axis. These layers are held together by weak van der Waals forces allowing intercalation of guest ions. Each layer comprises an alternating pair of edge-sharing and corner-sharing distorted VO_5_ square pyramids. Each VO_5_ unit has three inequivalent types of oxygen; one terminal oxygen center O1 forming a vanadyl bond V=O of 1.58 Å, one bridging oxygen center O2 connects to two adjacent V centers at V–O bond length of 1.78 Å, and three chain-forming oxygen centers O3 which are 3-fold coordinated with two V–O band lengths of 1.88 Å, and one with the bond distance of 2.02 Å (experimental values, Table S1 and Fig. S1†).

It can be seen from Table S1† that our calculated lattice parameters, *a* = 11.65 Å, *b* = 3.63 Å, *c* = 4.44 Å, and V–O bond distances, *d*_V–O1_ = 1.61 Å, *d*_V–O2_ = 1.81 Å, and *d*_V–O3_ = 1.91, 2.04 Å, provide good agreement with those of the experimental value^69^ and the previously calculated values.^54,55^ The optimized unit cell as just described was then used to construct a bulk model with 1 × 3 × 3 supercell of V_36_O_90_ for exploring the formation of an electron small polaron and its transport properties in the V_2_O_5_ lattice.

At the beginning of the discharge process, electrons flow from the current collector to the cathode while intercalation of Li^+^ ions occurs at the electrolyte–cathode interface to maintain the charge balance. It is evidenced that not only ion diffusion but also the electron transport plays an important role in determining the electrochemical kinetics at the cathode.^49,50^ Previous computational studies examined the behavior of polaron formation in proximity and relatively far from the intercalated ion in the V_2_O_5_ cathode in the context of Li-ion and Mg-ion batteries.^26,39^ In addition, several experimental and computational works have extensively been studied and reported evolution of atomic and electronic structures of various V_2_O_5_ phases upon lithiation in the context of cathode materials for Li-ion batteries.^26,27,38–41^

For the sake of completeness, in this work, we re-examined the atomic and electronic structures of polaron formation in V_2_O_5_ to ensure the validity of our computations. We considered the formation of an electron polaron in two different situations, *i.e.* when the electron is in the remote distance and in the vicinity of its Li-ion counterpart. The first situation happens at the very beginning of the discharge process. While electrons are first injected into the cathode, the Li^+^ ions start to intercalate into the cathode at the electrolyte–cathode interface. These extra electrons are still far from the electrolyte–cathode interface that their behaviors are unaffected by the intercalated Li^+^ ions. As the discharge progresses, electrons and Li^+^ ions travel to meet with one another, and the latter situation dominates. The behavior of the electrons is influenced by the presence of their counterparts.

**Fig. 1:**
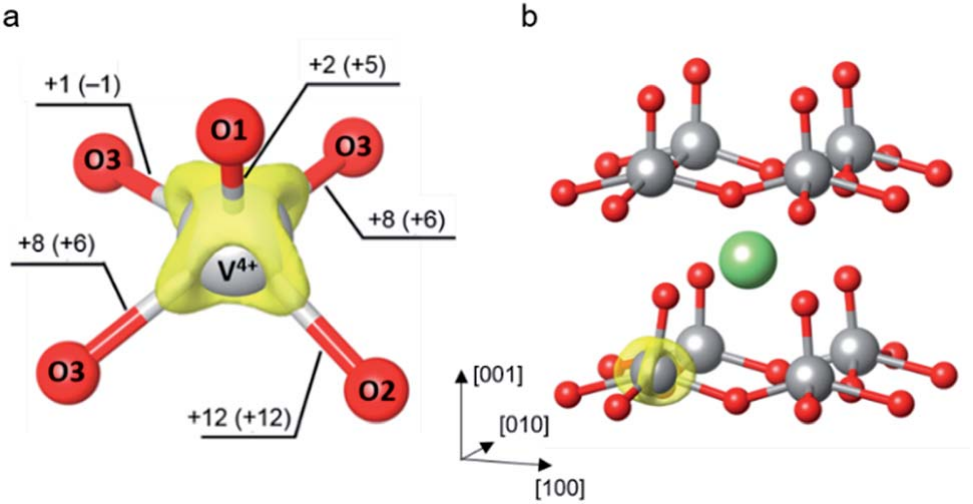
(a) Isosurface of the charge density difference between the polaronic supercell (including the electron-doped system and the Li-intercalated system) and the neutral supercell plotted at 0.01 e Å^−3^. The labelled numbers outside and inside the parentheses indicate changes of V–O bond distances (in pm) induced by the polaron formation in the electron-doped and Li-intercalated systems, respectively. (b) Schematic illustration of the most stable Li-intercalated site at the hollow site between the V_2_O_5_ layer. The isosurface of charge density difference indicates the reduced V center.

As of the first situation, electrons are far apart from Li^+^ ions, the formation of a small polaron in the absence of Li^+^ ions is of interest. An extra electron is added into the V_36_O_90_ supercell to obtain an electron-doped V_2_O_5_ system. The small perturbation to distort a VO_5_ unit was required to break the lattice symmetry which allows a proper localization of the added electron. The initial guess of the distorted VO_5_ square pyramid was taken from the reduced VO_5_ unit of the Li-intercalated system to mimic the structural reorganization occurred due to the electron localization. Then, we carried out a structural optimization to obtain the polaronic structure. The relaxed internal coordinates reveal the formation of an electron small polaron at the perturbed VO_5_ unit where the V center is reduced from V^5+^ to V^4+^. The polaron formation induces local structure distortion around the reduced V center. Major elongations of the V–O bonds are within the V_2_O_5_ layer in the *x*–*y* plane (Δ*d*_V–O2_ = 12 pm, Δ*d*_V–O3_ = 8 pm) while the bond lengthening in the *z*-direction is not as much (Δ*d*_V–O1_ = 3 pm), as shown in Fig. 1a. The isosurface of the positive part of differential charge density lies mostly along the V–O bonds within the layer which is in agreement with the structural changes that the in-plane lattice distortion is greater than that in the normal direction. The differential charge density was computed by subtracting the charge density of the polaronic system by that of the system without extra electrons. Both densities were calculated self-consistently with the same ionic coordinates.^28^ All isosurface and 3-dimensional representations of crystal structures were generated using VESTA.^70^ In addition, the calculated polaron formation energy reveals that the polaronic configuration is 0.40 eV more stable than the delocalized state at the *U* value of 4.0 eV, as detailed in ESI Section S2.†

In the latter situation, the Li^+^ ions and their electrons reconcile, we evaluated the polaronic structure of the lithiated V_2_O_5_. A Li atom was introduced into the supercell to yield a dilute Li concentration corresponding to Li_0.06_V_2_O_5_. As shown in Fig. 1b, the most stable intercalated site is at a hollow site above a ring formed by four VO_5_ units between V_2_O_5_ layers (see also ESI Section S3†). The lowest energy site is indeed the same as reported by other DFT studies.^25,26,39,54–57^ The ionized Li^+^ ion donates an extra electron to the lattice which localizes at the nearest V center and forms a small polaron. Note that no structural perturbation was made to obtain the polaronic structure since the Li^+^ ion induces small reorganization of the nearby oxygen atoms. It can be seen from Fig. 1 that the presence of the Li^+^ ion has only small effects to the polaronic structure as it exhibits similar V–O bond elongations of the reduced VO_5_ square pyramid. The most pronounced contribution is observed at the slightly larger elongation of V–O1 bond (Δ*d*_V–O1_ = 5 pm) where the terminal oxygen is pulled toward the positively charged Li^+^ ion. In addition, its isosurface of charge density difference is very similar to that of the electron-doped system that the charge difference density lies mostly along the elongated V–O bonds in the *x*–*y* plane. Similar observation on structural reorganization and charge localization upon a polaron formation in V_2_O_5_ was also reported in previous computational works using the DFT+U method.^25–27,39,42,56,71^

**Fig. 2:**
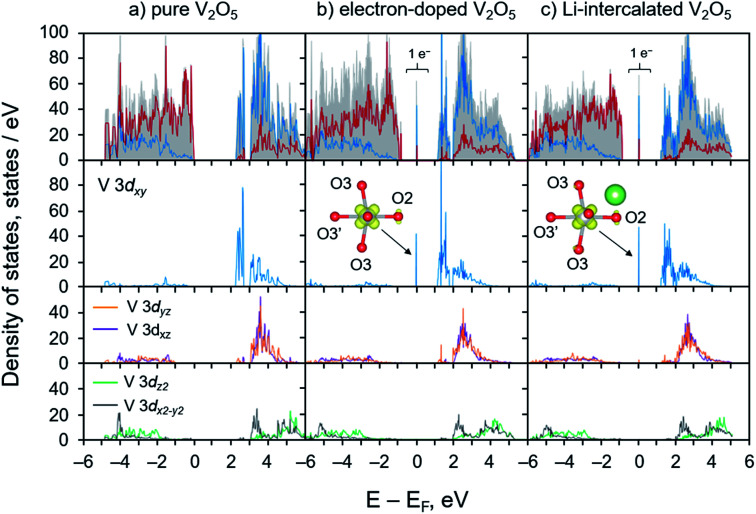
Projected density of states of (a) pure V_2_O_5_, (b) electron-doped V_2_O_5_, and (c) Li-intercalated V_2_O_5_ systems. In the topmost panel, red and blue lines represent states contributed by O 2p and V 3d, respectively. The shaded gray area illustrates the total density of states. The gap states appeared in panel (b) and (c) are accounted for 1 electron per supercell which mainly contributed by V 3d_*xy*_. Their isosurface obtained from band decompose charge calculations are plotted in the insets.

We calculated and analyzed the PDOS of the electron-doped and the lithiated V_2_O_5_ systems and compared to that of bulk V_2_O_5_. As depicted in Fig. 2a, the PDOS of V_2_O_5_ exhibits semiconducting character with a calculated band gap of 2.26 eV, which is consistent with the other work employing the PBE+U method^54^ and agrees well with the experimental band gap of 2.3 eV.^72^ The valence band mainly comprises O 2p states with some contributions from V 3d states. The conduction band exhibits a unique split-off band character due to the strong deviations of the distorted VO_5_ square pyramids from their cubic symmetry, the VO_6_ octahedra.^73^ Such distortions reduce the local symmetry at the V sites to monoclinic which dramatically affect d orbital splitting of octahedral crystal field (t_2g_–e_g_ configuration). The low-lying conduction band is mainly composed of V 3d_*xy*_ states in the energy range from 2.46 to 2.57 eV. Across a small gap of 0.33 eV, V 3d_*yz*_ and 3d_*xz*_ states equally contribute to the total DOS in the energy range between 2.9 and 5.0 eV whereas the e_g_ states, V 3d_*z*^2^_ and 3d_*x*^2^−*y*^2^_, dominate at higher energies (4.5 to 6.0 eV). These bands are predominantly contributed by V 3d states with the non-negligible mixing of O 2p states. Our calculated results are consistent with those obtained from the recent studies that the character of split-off conduction band has been identified through an interpretation of X-ray absorption near-edge structure (XANES) spectra and time-dependent DFT calculations.^38,74^

The electronic structures (PDOS) of the electron-doped and the lithiated systems are very similar. Their key features of the polaronic states are identical as illustrated through the position of the localized gap state of 1 electron at the V 3d_*xy*_, Fig. 2 panel b and c. The deep localized state is due to local trapping of the excess electron at the reduced V center as illustrated *via* elongations of V–O bonds of the reduced VO_5_ unit, Fig. 1. PDOS analysis and band decompose charge density calculations indicate that most of the excess electron density tends to occupy the empty low-lying V 3d_*xy*_ state of the distorted octahedral crystal field, and only a small fraction is on the surrounding five O atoms, as shown in Fig. 2 panel b and c and the insets. Overall, the calculated electronic structures agree very well with the observed geometrical changes due to the polaron formation. The self-trapped electron occupying the V 3d_*xy*_ state leads to major elongations of V–O bonds in the *x*–*y* plane within the V_2_O_5_ layer. These calculated results are consistent with the earlier DFT works and experimental findings that the formation of a small polaron induces structural reorganization in several semiconducting materials including V_2_O_5_,^25,26,39,54,56,75^ TiO_2_,^76–78^ Fe_2_O_3_,^79^ BiVO_4_,^80,81^ and Li_*x*_FePO_4_.^28^

Although this work only focuses on the intercalation of Li in the α-V_2_O_5_ structure, it is worth noting that higher degree of lithiation induces phase transformation of Li_*x*_V_2_O_5_ structures which leads to changes in their corresponding electronic structures. As illustrated through XANES spectra, scanning transmission X-ray microscopy images, and first-principles computations, increase of Li concentrations brings about further distortion of the square pyramids away from octahedral symmetry resulting in the increased interlayer separation and disrupt the long V–O interactions between layers.^38,71^ The lithiation and concomitant reduction of V_2_O_5_ was observed *via* the modification of XANES spectra that the V 3d_*xy*_ states at the split-off conduction band is strongly diminished as a result of localized polaron states.^38,71,82^

### 3.2 Electron transport properties in bulk V_2_O_5_

A self-trapped polaron at a reduced V center can undergo thermally activated hopping from one localized well to another throughout the lattice. The mobility of electron polaron in bulk V_2_O_5_ depends on its local migration barrier between two equilibrium polaronic sites. We explored various possible migration paths in the stoichiometric V_2_O_5_ and calculate their energy barriers. As described in the computational details, we employed two methods to calculate the migration barriers of polaron migration, namely, linear interpolation (LE) and NEB methods.

**Fig. 3:**
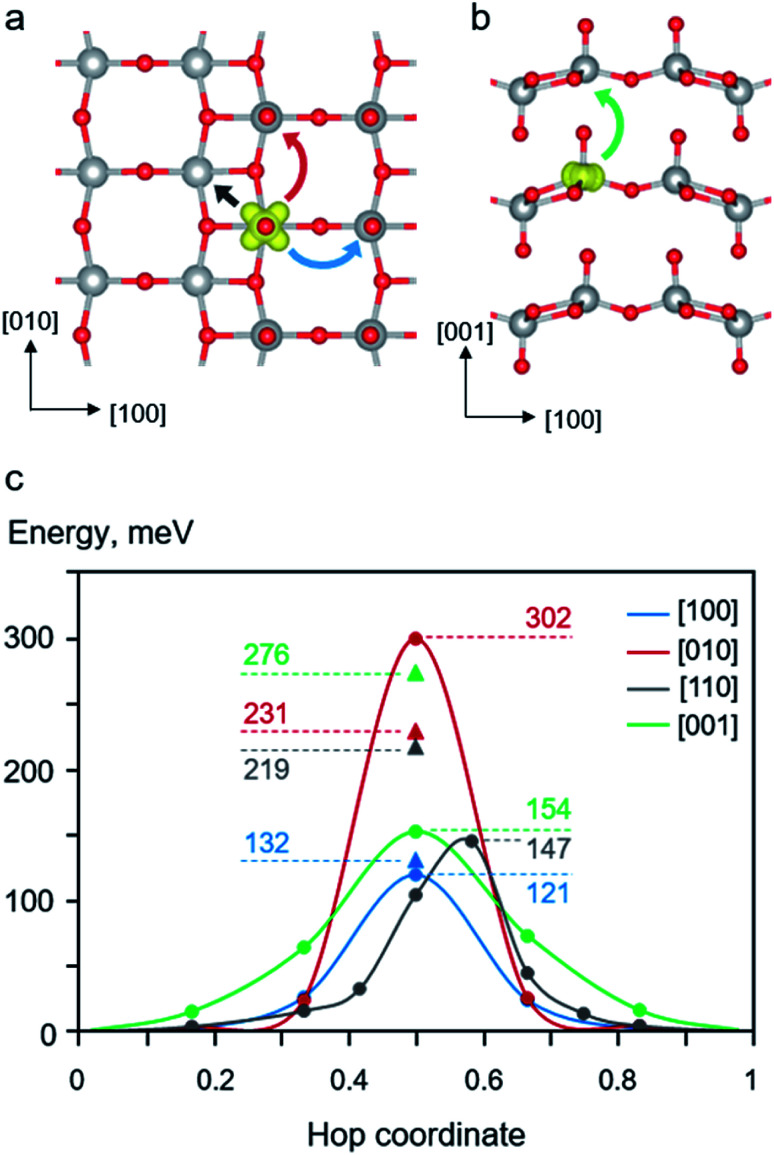
Schematic illustrations of polaron migration paths for (a) intra-layer hopping and (b) inter-layer hopping. The blue, red, black, and green curve arrows represent polaron hopping along [100], [110], [010], and [001] directions, respectively. (c) Potential energy profiles of polaron migration along different crystallographic directions. The LE-calculated barriers are marked as solid triangles where the NEB-calculated energy profiles are also plotted. The labelled numbers are the calculated barriers in meV.

We first examined the behavior of polaron migration in the absence of Li^+^ ions using the LE method. A series of linearly interpolated structures between two adjacent polaronic sites were created. Their calculated single-point energies were used to determine the migration barriers. As schematically depicted in Fig. 3 panel a and b, we consider four inequivalent migration paths including movements along the [100], [010], [110], and [001] directions. The calculated results reveal anisotropic hopping barriers along different crystallographic directions. As shown in Fig. 3c, the migration along the [001] direction exhibits the highest barrier of 276 meV because the polaron needs to hop across the van der Waals gap with the longest hopping distance between of 444 pm. In addition, its transition state (TS) structure displays disconnected V*–O1–V* bonds along the [001] direction. The relatively large separation and disconnected bonds between two V centers result in a small overlap between two polaronic states at the TS.

Lower migration barriers were predicted for polaron hopping within the V_2_O_5_ layer along the [100] (*E*_a_ = 132 meV), [010] (*E*_a_ = 231 meV), and [110] (*E*_a_ = 219 meV) directions. Such behavior of polaron movement can be explained in terms of different TS structures along the migration paths. In contrast to the [001] migration, the in-plane movements involve shorter hopping distances where all TSs display connected V–O–V bonds between two polaronic sites. Nevertheless, the movement in the [010] direction is kinetically less favorable than those of the other in-plane directions because the hopping distance is quite large (364 pm). In addition, the TS structure predominantly involves a displacement of the stiff 3-coordinated oxygen atom (O3) connected between two V centers in the [010] direction resulting in a distorted TS structure and high energy. The same is true for the migration along the [110] direction where the two V centers are connected *via* two O3 atoms. On the other hand, the movement along the [100] direction exhibits the lowest barrier of 132 meV even though the hopping distance is quite large, 354 pm. This is because the TS structure mostly contains a displacement of a relatively more flexible bridging oxygen atom (O2) leading to a less constrained TS structure and a lower TS energy. The structural reorganization accompanying a supple movement of O2 atom facilitates the polaron migration along the [100] direction. The calculated barriers are consistent with other computed barriers in [010] direction at PBE+U level, Li_0.06_V_2_O_5_ (0.28 eV)^25^ and Li_0.083_V_2_O_5_ (0.34 eV),^39^ and experimentally obtained values for single crystals (0.27 eV),^83^ nanorods (0.23 eV),^84^ and thin films (0.28 eV).^85^ In addition, the effect of *U* parameter on the computed migration barriers is discussed in the ESI Section S4.†

Next, configurations along the migration paths generated from the LE approach were optimized using the NEB method. As shown in Fig. 3c, the NEB calculated barriers (for [100], [001], and [110] in the range of 121–154 meV) are generally lower than those obtained from the LE calculations (132–176 meV). This is expected because the NEB computations allow for structural optimization of configurations along the MEP resulting in numerical variation of the calculated barriers. Interestingly, the used NEB method predicts significantly higher hopping barrier than that of the LE method for the [010] migration (302 *vs.* 276 meV). The origin of the energy difference can be explained through their TS structures. As discussed above, the [010] hopping exhibits a rather high barrier due to the displacement of the rigid O3 atom connected between two V centers along the [010] direction. The NEB-optimized configuration yields an even more strained TS structure than that obtained from the LE method leading to the higher TS energy as discussed in the ESI Section S5.† The NEB-TS structure involves a relatively short V*–O3 bond distances and smaller V–O3–V bond angles. Such a structure with shorter V–O2 bonds is not preferred to accommodate a small polaron resulting in a relatively high energy. On the contrary, the calculated NEB barrier for [001] hopping is dramatically lower than that calculated using the LE method (154 *vs.* 276). The LE method predicts a relatively high TS energy since the distance between the two V centers along the [001] direction is quite large and that the TS exhibits disconnected V*–O1–V* bonds with the O1–V* distance across the van der Waals gap of 272 pm. Using the NEB method results in a more relaxed TS structure with the shorter O1–V* distance of 269 pm leading to a lower TS energy, as discussed in ESI Section S5.†

Our calculations employed the DFT+U method which implies the validity of Born–Oppenheimer approximation to calculate the adiabatic activation energy. The calculated potential energy profiles were derived from linear interpolation (LE) and optimization of ionic coordinates along the migration path (NEB) where their energies were computed self-consistently. The adiabaticity of the polaron transport can be determined by computing the electronic coupling constant (*V*_AB_) as described in the computational details.^68^ Computations show that the polaron transfer in all considered directions, in the absence of the Li^+^ ion, are merely non-adiabatic. As described in ESI Section S6,† our calculated *V*_AB_ values are negligible where the DOS of TS structures exhibit a single gap state between valence band and conduction band. This is because the hopping distances between two V centers are quite large where the shortest distance, 313 pm, belongs to the [110] hopping. The predicted behavior of non-adiabatic hopping is consistent with experimental observations that the polaron hopping in V_2_O_5_ glass and thin film are in the non-adiabatic regime.^86,87^

Based on the LE computations, it can be seen that the interlayer hopping displays a somewhat higher barrier than those of the in-plane migrations. The in-plane conduction can be described by continuous movement of polaron along two perpendicular directions, [100] and [010], throughout the lattice. The conduction along the [100] direction includes a series of [100] and [110] hopping where the latter exhibits a higher barrier of 219 meV and therefore is the rate-limiting step. The [010] conduction can undergo in two different pathways, *i.e.*, through the [010] hopping or a series of [110] movements. It can be seen that the zigzag [110] hopping is kinetically more favorable (219 *vs.* 231 meV), thus, it is the preferred migration path. Considering all possible migration paths, the in-plane conductivity is limited by the movement in the [110] direction (*E*_a_ = 219 meV) which is significantly lower than the hopping barrier of the normal direction (276 meV) resulting in anisotropic polaron conduction. We can roughly estimate polaron mobilities within and across the V_2_O_5_ layer by calculating the hop frequency as *ν* = ν* exp(−*E*_a_/*k*_B_*T*), where ν* is the attempt frequencies assumed to be very similar for all considered paths. At room temperature, computations predict that the polaron mobility within the layer is approximately one order of magnitude higher than that perpendicular to the layer, due to the high migration barrier across the van der Waals gap. Interestingly, the estimates agree very well with the measured electronic conductivity in the single crystal V_2_O_5_ at room temperature that the in-plane conductivity (10^−3^ S cm^−1^) is also one order of magnitude greater than the normal (10^−4^ S cm^−1^).^83^ Our computations, based on the LE-calculated barriers, are in agreement with the previously reported experimental observations^83^ and suggest that it is kinetically more favorable for electron polarons to travel within the V_2_O_5_ layer than that across the van der Waals gap.

Considering the in-plane migration, the NEB computations also display a similar trend to those of the LE calculations that the [010] hopping in kinetically least favorable. The NEB-calculated barrier of [010] hopping is 302 meV while the [100] and [010] migrations exhibit barriers of 121 and 147 meV, respectively. Nevertheless, the NEB method describes the in-plane and normal conduction quite differently. As discussed above, the in-plane conduction, limited by the [110] hopping, possesses a barrier of 147 meV which is slightly lower than the hopping in the normal direction (154 meV). Using the formula described above, the polaron mobilities within the V_2_O_5_ layer and that across the plane are very similar. It is very interesting to find that although the NEB method allows for structural relaxation of configurations along the migration path where more accurate TS configurations and energy barriers are expected, the LE method provides a better agreement with the experimental observations in terms of anisotropic polaron mobilities.

### 3.3 Effect of Li intercalation on the behavior of electron transport

**Fig. 4:**
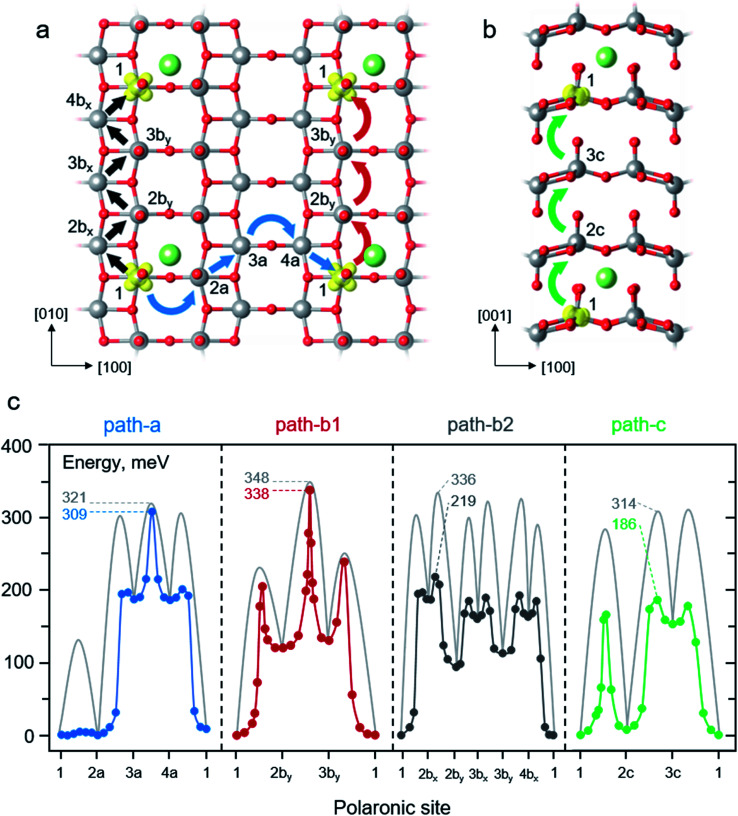
Schematic illustrations of polaron migrations along different crystallographic directions within the layer (a) and across the layer (b). The blue, red, black, and green curve arrows represent polaron hopping in path-a, path-b1, path-b2 and path-c, respectively. The labelled numbers indicate different polaronic sites along the polaron migration paths. (c) Potential energy profiles of polaron hopping corresponding to the paths described in panel a and b. The NEB-calculated MEPs are shown in blue, red, dark grey, and green lines for polaron hopping in the path-a, path-b1, path-b2 and path-c, respectively. The light grey lines represent calculated MEPs using the LE method. The labelled numbers indicate the effective barriers of each path in meV. The corresponding reaction energies and energy barriers of all steps are reported in Table 1.

As the discharge progresses, the electrons travel through the cathode and finally reunite with the Li^+^ ions. At such a situation, the electron transport properties at dilute Li concentrations is considered. The effect of lithiation to transport properties of polaron is of interest. Thus, we examined the migration of a small polaron in the presence of a Li^+^ ion in the 1 × 3 × 3 V_2_O_5_ supercell. As schematically depicted in Fig. 4 panel a and b, four different migration paths were considered according to crystallographic directions including a combination of [100], [110], and [11̄0] directions (path-a); the [010] direction (path-b1); a combination of [110] and [1̄10] directions (path-b2); and the [001] direction (path-c). The potential energy profiles were computed considering series of polaron movements between two V centers along a migration path at a fixed Li-ion position. The hopping continues until the electron reconciles with the Li^+^ ion of an adjacent periodic image. For example, polaron movement in path-a begins with a hopping from the polaronic site 1 to 2a (1 → 2a) in the [100] direction followed by 2a → 3a and 3a → 4a in the [110] and [100] directions, respectively. Finally, the movement of 4a → 1 in the [11̄0] direction yields an equivalent polaronic state 1 of the starting structure and completes the migration cycle. The calculated energy profiles along each migration path using LE and NEB methods are plotted in Fig. 4c where their reaction energies and migration barriers are summarized in Table 1.

**Table 1:** Calculated reaction energies, *E*_r_, and barriers, *E*_a_, (both in meV) of polaron migrations in all considered paths of the Li-intercalated V_2_O_5_ system using LE and NEB methods

Path	*E*_r_	*E*_a_	
		LE	NEB
**Path-a**			
1 → 2a	1	135	4
2a → 3a	188	302	195
3a → 4a	−1	132	122
4a → 1	−180	119	15

**Path-b1**			
1 → 2b_*y*_	120	235	205
2b_*y*_ → 3b_*y*_	10	228	218
3b_*y*_ → 1	−130	121	109

**Path-b2**			
1 → 2b_*x*_	188	302	196
2b_*x*_ → 2b_*y*_	−67	148	21
2b_*y*_ → 3b_*x*_	40	180	88
3b_*x*_ → 3b_*y*_	−30	162	29
3b_*y*_ → 4b_*x*_	33	193	75
4b_*x*_ → 1	−164	123	21

**Path-c**			
1 → 2c	7	284	166
2c → 3c	146	302	179
3c → 1	−153	161	25

We first discuss the behavior of the polaron hopping along the path-a. It begins with a hopping from configuration 1 to 2a, 1 → 2a, in the vicinity of the Li^+^ ion. As depicted in Fig. 4c and summarized in Table 1, the polaron hopping is considered an isothermal change with the reaction energy of 1 meV due to the slightly off centro-symmetric position of the Li^+^ ion (see also ESI Section S3 and Fig. S3b†). As for the energy profile calculated using the LE method, the 1 → 2a hopping features a relatively small barrier of 135 meV which is very similar to that of the polaron migration along the [100] direction in pure V_2_O_5_ (132 meV). Its TS structure mostly involves a displacement of a flexible O2 atom in the vicinity of a fixed Li^+^ ion, thus, the barrier is quite similar to that of the migration in the absence of the Li^+^ ion. Followed by the 2a → 3a hopping along the [110] direction, the polaron moves away from its Li-ion counterpart leading to an endothermic process of 188 meV. Such a movement yields significantly greater migration barrier (302 meV) than that of the hopping in the absence of Li^+^ ions (219 meV) in the same crystallographic direction. The origin of such a high barrier can be explained that the TS structure involves displacement of stiff O3 atoms (as previously discussed) with an increasing ion-polaron separation as the polaron is being moved from site 2a to 3a. The subsequent step, 3a → 4a, is the hopping in the absence of the adjacent Li^+^ ion. This process is considered isothermal since the energy difference between the product state and the reactant state is as small as 1 meV. Its migration barrier, 132 meV, is equivalent to that of the migration in pure V_2_O_5_ along the [100] direction. The final step, 4a → 1, involves the polaron hopping toward the Li^+^ ion. The hopping overcomes a rather small barrier of 119 meV and gives a more stabilized state by 188 meV. This step can be viewed as the reverse process of the 2a → 3a where they share the same TS structure and energy. As shown in Fig. 4c, the NEB-calculated energy profiles exhibit similar features to that obtained from the LE calculations because they share the same intermediates along the migration path. However, using the NEB method results in significantly lower hopping barriers for the steps involving polaron hopping nearby the Li^+^ ion, *i.e.*, 1 → 2a, 2a → 3a, and 4a → 1 (Table 1). This is because the TS structures obtained from the NEB calculations are allowed to relax to incorporate the presence of the adjacent Li^+^ ion while the LE-calculated TS structures are restricted to the LE-generated configurations. Nevertheless, the LE- and NEB-calculated barriers are very similar for the 3a → 4a migration (132 *vs.* 122 meV) as also observed for the hopping in bulk V_2_O_5_. Overall, due to the highly endothermic reaction of 2a → 3a, the effective barriers of the polaron migration in the path-a are calculated to be 321 meV and 309 meV for LE and NEB computations, respectively.

Next, we examined the polaron migration along the path-b1. The starting configuration 1 involves a polaronic site at the reduced V center (V1) closest to the inserted Li^+^ ion at the Li–V1 distance of 311 pm which is smaller than that of the Li–V2b_*y*_ distance (382 pm) of configuration 2b_*y*_ (see also ESI Section S3 and Fig. S3b†). In this first step, 1 → 2b_*y*_, the polaron undergoes an endothermic movement of 120 meV where the product state 2b_*y*_ exhibits a higher energy configuration as a result of a larger Li–polaron separation. Its LE-calculated barrier (235 meV) is comparable to that of the hopping in the pure V_2_O_5_ (231 meV) as their TS structures are very similar with only small differences of the average V–O bond (less than 2%) due to the presence of Li^+^ ion in the latter calculation. As shown in Table 1, the NEB-calculated barrier of the 1 → 2b_*y*_ hopping (205 meV) is lower than that of the LE value (235 meV) due to the more relaxed TS structure in the presence of the nearby Li^+^ ion. Subsequently, the 2b_*y*_ → 3b_*y*_ hopping takes place in the absence of nearby Li^+^ ions. As expected, its reaction energy (10 meV) and the LE-calculated barrier (228 meV) are comparable to those of the hopping in the pure V_2_O_5_ along the [010] direction (isothermal, *E*_a_ = 231 meV). The similar hopping barriers are due to the resemblance of their TS structures where the difference of average V–O bond distance is less than 2%. The NEB calculations describe the 2b_*y*_ → 3b_*y*_ hopping quite similarly as the calculated barrier (218 meV) is slightly lower than that of the LE method (228 meV) as shown in Table 1. At the last step, the polaron hops from the configuration 3b_*y*_ to 1, 3b_*y*_ → 1, to reassemble its periodic image. Hence, the final step exhibits an exothermic change of 130 meV with a LE-hopping barrier of 121 meV. The TS energy is considerably high due to the strain occurred from the movement of a stiff O3 atom along the [010] direction. A more relaxed TS structure obtained from the NEB calculation leads to a slightly lower barrier of 109 meV. Nevertheless, the movement of the negatively-charged polaron toward the positively-charge ion lowers the total energy resulting in the relatively low migration barrier. In conclusion, polaron migration in the path-b1 exhibits the highest effective barriers for both LE (348 meV) and NEB (338 meV) calculations due to the strained TS structures occurred from O3 displacement along the migration path. Our calculated barriers along the path-b agree very well with previously calculated values using the PBE+U method with *U*_3d_ (V) = 4.0 eV (*E*_a_ = 0.31 eV)^25^ and *U*_3d_ (V) = 3.1 eV (*E*_a_ = 0.34 eV).^26^

Alternatively, the [010] migration can be assembled from consecutive movements in the [110] and [1̄10] directions as described in the path-b2. The migration involved series of zigzag movements starts off with the 1 → 2b_*x*_ hopping which exhibits an endothermic change of 188 meV (Table 1). The LE method predicts a rather high barrier of 302 meV while the NEB computations provide a much lower barrier of 196 meV because the NEB-calculated TS structure can be relaxed to incorporate the presence of nearby Li^+^ ion (Table 1). Note that the polaron hopping 1 → 2b_*x*_ is essentially the same movement as 2a → 3a, hence, its TS structure and energy are the same as those of the 1 → 2b_*x*_ hopping. The subsequent movement, 2b_*x*_ → 2b_*y*_, overcomes a LE (NEB) barrier of 148 (32) meV and releases energy by 67 meV as indicated in Table 1. This step has the TS structure and energy analogous to that of the polaron movement in the absence of Li^+^ ion along the [110] direction where the difference of average V–O bond distance is less than 2% for both LE and NEB calculations. The exothermicity due to the polaron-ion attraction significantly reduces the hopping barrier. Combining the 2 movements, 1 → 2b_*x*_ and 2b_*x*_ → 2b_*y*_, gives rise to the one step hopping of 1 → 2b_*y*_ in the path-b1. It can be seen that, for the LE computations, the 2-step movement of path-b2 displays considerably higher effective barrier (336 meV) than that of the 1-step hopping of the 1 → 2b_*y*_ (235 meV) of path-b1 as shown in Fig. 4c and Table 1. On the other hand, the NEB-calculated effective barriers of the combined 2-step (219 meV) and the 1-step movement (205 meV) are quite similar (Fig. 4c and Table 1). The subsequent steps along the migration path display lower TS energies than the 2b_*x*_ → 2b_*y*_ hopping; as a result, the effective migration barriers of 336 meV and 219 meV are determined using the LE and NEB methods, respectively. Overall, as predicted by the LE calculations, the effective migration barriers of path-b1 and path-b2 are quite similar. On the contrary, the NEB-calculated energy profiles reveal that it is kinetically more favorable for polaron transport along the path-b2 (219 *vs.* 338 meV).

Lastly, the behavior of polaron hopping along the path-c is explained. The first step, 1 → 2c, involves the polaron migration in proximity with the Li^+^ ion across the van der Waals gap. Due to the similar Li-polaron distances of configuration 1 (318 pm) and 2c (307 pm), the 1 → 2c hopping is endothermic by 7 meV. We note here that even though the ion-polaron distance of the configuration 1 is larger, it displays a lower energy intermediate because of the coulombic attraction between the Li^+^ ion and the negatively-charged O1 of the configuration 1. As shown in Fig. 4c and Table 1, the LE-calculated migration barrier (284 meV) is, to some extent, higher than that of the inter-layer hopping in the pure V_2_O_5_ (276 meV). Similarly, the NEB computations reveal that the polaron hopping in the vicinity of the Li^+^ ion (166 meV) is somewhat higher than the bulk migration in the same direction (154 meV). The attraction between negatively-charged oxygen ions around the Li^+^ ion leads to stretched V–O bonds resulting in the strained TS geometry with higher TS energy. Next, the 2c → 3c hopping occurs as the polaron moves further away from the nearest neighbor Li^+^ ion leading to an endothermic change of 146 meV with a rather high migration barrier of 302 and 179 meV for LE and NEB calculations, respectively (Table 1). The calculated TS energies are relatively higher than those of the bulk migration (Fig. 3c) in the same direction for both LE (276 meV) and NEB (154 meV). Such unstable TS structures stem from the increased ion-polaron separation at the TS configuration. The final step, 3c → 1, features a highly exothermic process (−153 meV) and relatively low LE- and NEB-calculated barriers of 161 and 25 meV, respectively (Table 1). The release in energy with a low hopping barrier is expected since the polaron moves toward the Li^+^ ion. To sum up, the LE and NEB computations display a large difference of effective barriers (314 *vs.* 186 meV). The barriers obtained from the NEB calculations are significantly lower due to the more relaxed TS structures that allow the movement of O1 towards the other V atom across the van der Waals gap.

It can be seen from the calculated results that there are numerical variations between the LE- and NEB-calculated barriers. As previously discussed, the origin of the difference stems from the fact that the NEB calculations allow for structural optimization of configurations along the migration paths. Consequently, the more stable TS structures and energies are predicted when the NEB method is used. Similar predictions have been reported in many computational works that the NEB-calculated barriers are generally lower than those calculated using the LE method as found in MoO_3_,^88^ BiVO_4_,^89,90^ and Fe_2_O_3_.^68^ Nevertheless, both LE and NEB computations indicate that the presence of the Li^+^ ion dramatically affects the behavior of polaron transport in the V_2_O_5_ cathode due to the strong ion-polaron attraction. The ion-polaron interaction can be estimated by comparing the reaction energies of the hopping step involving the nearest neighbor Li^+^ ion. As summarized in Table 1, the polaron hopping in the path-a and path-b1 exhibits the largest ion-polaron interaction as the reaction energy of 2a → 3a (also 1 → 2b_*x*_, 188 meV) is higher than those of the movements in the path-b1 (1 → 2b_*y*_, 120 meV) and the path-c (2c → 3c, 146 meV). The presence of a Li^+^ ion also affects the polaron migration barriers. The polaron hopping in the vicinity of a Li^+^ ion generally displays higher barriers than those of the migration in the absence of the nearest Li^+^ ion along the same direction. The increased migration barriers and the relatively high intermediate energies due to the strong ion-polaron interactions lead to considerably higher effective barriers of polaron migration in all crystallographic directions. The estimated hop frequencies reveal that the polaron mobility is diminished by up to three order of magnitude when the polaron hops within proximity of the Li^+^ ion.

In addition, the adiabaticity of polaron hopping in the presence of the intercalated Li^+^ ion was determined. Computations reveal that lithiation does not affect the adiabaticity of polaron migration in the V_2_O_5_ lattice. Polaron hopping in every step in all considered path are non-adiabatic as the calculated *V*_AB_ determined from the gap states of TS configurations are negligible. As shown in ESI Section S6,† PDOS of all TS configurations display a single gap state. Our prediction is consistent with the previous computational study that the polaron transport in the Li-intercalated V_2_O_5_ along the [010] direction is non-adiabatic.^25^ Their calculated *V*_AB_ obtained using the simplified fragment charge difference method^91^ is very small, *V*_AB_ = 0.001 eV. Moreover, the predicted behavior of polaron hopping is in agreement with the experimental observation that the conduction mechanism of lithiated V_2_O_5_ xerogel obeys non-adiabatic small polaron hopping.^92^

Note that several computational works used various methods to determine the character of polaron transport in transition metal oxides (TMOs). While the hybrid functional based methods are more robust and consistent, its computational demanding forbids us to apply the method to a relatively large supercell. The DFT+U approaches, with appropriate *U* values, have been demonstrated to be an effective method to studying polaronic properties in several TMOs such as TiO_2_,^93,94^ Fe_2_O_3_,^68^ MnO_2_,^95^ and LiFePO_4_.^95,96^ In particular, the polaron hopping barriers calculated using the DFT+U methods were compared with the hybrid functional methods where many studies reported reasonable agreement in the calculated barriers in several TMOs such as BiVO_4_,^89,97^ MoO_3_,^88^ and CeO_2_.^98^

The computations suggest that once the polaron meets with its Li-ion counterpart, its kinetics becomes very sluggish that it could be the rate-limiting step of the charge transport. Nevertheless, other diffusion events, such as ion diffusion and coupled polaron-ion diffusion, may also play a role in determining the diffusion kinetics. As reported in the recent computational study using PBE+U (*U*_3d_ (V) = 4.0 eV), the calculated apparent activation barrier of the coupled polaron-ion diffusion is in the range of 0.3 eV.^25^ which is comparable to our calculated polaron migration barriers, ∼0.3 eV. In addition, our calculated results reveal that polaron hopping in the [010] direction exhibits the slowest kinetics which contrasts with the ion diffusion where the [010] diffusion is the most favorable path.^25^ Nanostructured design to shorten the carrier migration path, in particular, along the [010] direction, or doping with foreign elements to increase charge carriers and alter VO_5_ polyhedra could effectively promote ion-polaron transport in the V_2_O_5_ cathode. Other studies also reported the behavior of ion-polaron diffusion in several oxide-based cathode materials, such as Li_*x*_V_2_O_5_,^25–27^ Li_*x*_FePO_4_,^28^ Li in TiO_2_,^29,30^ and Li_2_FeSiO_4_.^31^ In addition, at higher Li concentration, Li_*x*_V_2_O_5_ undergoes phase transformation where the limitations of transport kinetics were identified for ion diffusion at the phase boundaries.^40^

## 4 Conclusions

We carried out first-principles calculations to study electron transport properties in the V_2_O_5_ cathode of Li-ion batteries. We ensure that the employed method based on plane-wave DFT+U calculations properly describe the behavior of polaron formation in the V_2_O_5_ cathode as inspected through structural distortion and localization of electronic states. The behavior of polaron migration in the V_2_O_5_ cathode was explored using LE and NEB methods. Computations reveal that the NEB-calculated barriers are generally lower than those obtained from the LE calculations. This is because the NEB method allows for structural optimization of configurations along the path leading to more relaxed TS structures and lower TS energies. Nevertheless, both methods provide consistent predictions that the movement along the [010] direction is kinetically least favorable since it is hindered by the displacement of rigid O3 atoms. In addition, polaron mobilities along different crystallographic directions were estimated. The LE calculations predict anisotropic mobilities where the intra-layer conductivity is superior than that of the inter-layer conduction. The estimates are consistent with the anisotropic conductivity observed in experiments. Nevertheless, the NEB computations suggest a different scenario where the polaron mobilities within and across the layers are comparable. Effect of Li intercalation on the behavior of polaron migration was further explored. Electrostatic attraction between the negatively-charged polaron and the Li^+^ ion increases reaction energies and barriers of polaron hopping which in turn diminishes the charge transport kinetics of the V_2_O_5_ cathode. Furthermore, computations predict that the polaron hopping in the V_2_O_5_ lattice, in the presence and absence of the Li^+^ ion, is non-adiabatic which is consistent with several experimental observations. It is worth noting that the conclusions made in this study based on the situation of early discharge which involves dilute Li concentrations at the cathode. Behavior of charge transport is expected to change when the discharge progresses as the Li concentration increases leading to phase transformation of Li_*x*_V_2_O_5_.

## Conflicts of interest

There are no conflicts to declare.

## Supplementary Material

RA-009-C9RA02923K-s001
