# Supplementary material for: Transport properties of electron small polarons in a V2O5 cathode of Li-ion batteries: a computational study
Source: RSC Adv. 2019 Jun 21;9(34):19483–94. doi: 10.1039/c9ra02923k (PMC9065376; doi:10.1039/c9ra02923k)
Supplement: RA-009-C9RA02923K-s001 [file RA-009-C9RA02923K-s001.pdf]

Electronic Supplementary Material (ESI) for RSC Advances.
This journal is © The Royal Society of Chemistry 2019

Electronic Supplementary Information

Behavior of Electron Small Polarons in a V_2O_5 Cathode of Li-ion Batteries: A Computational Study

Panuwat Watthaisong,[a,b] Sirichok Jungthawan,[b,c,d] Pussana Hirunsit,[e] Suwit Suthirakun*,[a,b]

[a]School of Chemistry, Institute of Science, Suranaree University of Technology, Nakhon Ratchasima, Thailand 30000

[b]Center of Excellence in Advanced Functional Materials, Suranaree University of Technology, Nakhon Ratchasima, Thailand 30000

[c]School of Physics, Institute of Science, Suranaree University of Technology, Nakhon Ratchasima, Thailand 30000

[d]Thailand Center of Excellence in Physics (ThEP), Commission on Higher Education, 328 Si Ayutthaya Road, Bangkok, Thailand 10400

[e]National Nanotechnology Center (NANOTEC), National Science and Technology Development Agency (NSTDA), 111 Thailand Science Park, Pathum Thani, Thailand 12120

Content

Section S1. Structure of the V_2O_5 unit cell 2
Section S2. Formation of a small polaron in V_2O_5 3
Section S3. Position of the Li^+ ion in the V_2O_5 lattice 4
Section S4. Polaron hopping barriers as a function of U value 6
Section S5. Transition state structures obtained from LE and NEB calculations........ 7
Section S6. Estimation of electronic coupling constant........ 8
References 9

*Corresponding author. Email: suthirak@sut.ac.th

Section S1. Structure of the V_2O_5 unit cell

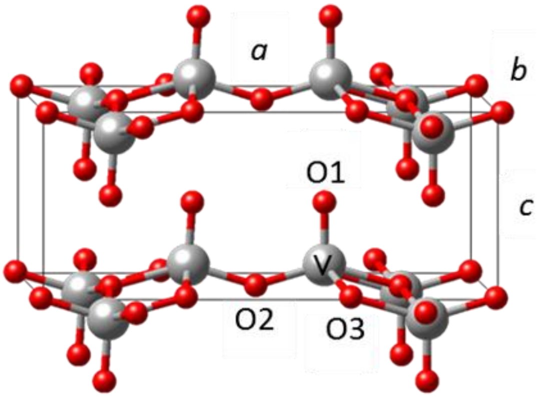

Fig. S1 Sketch of the crystal structure of the α-V_2O_5 unit cell where *a*, *b*, and *c* label the lattice vectors. Three types of oxygen centers were indicated as follow; O1 = terminal oxygen center, O2 = 2-fold coordinated oxygen center, and O3 = 3-fold coordinated oxygen center.

Table S1 Calculated Lattice Parameters and V−O Bond Lengths (both in pm) of Bulk V_2O_5, Using the optPBE+U-vdw Functional Compared to Available Experimental Values and Computed Values Using the PBE+U, and optPBE+U-vdW Functionals

	optPBE+U-vdw (this work)	PBE+U[a]	PBE+U[b]	optPBE+U-vdW[b]	Exp[c]
a	1167	1150	1157	1169	1151
b	363	363	363	363	356
c	440	480	468	442	137
$d_{V\text{-}O1}$	161	160	161	162	158
$d_{V\text{-}O2}$	181	181	181	181	178
$d_{V\text{-}O3}$	191	192	191	191	188
	204	202	203	205	202

[a]Ref [1], [b]Ref [2], [c]Ref [3]

Section S2. Formation of a small polaron in V_2O_5

Stability of polaron in the V_2O_5 lattice was examined by calculating energy of polaron formation as $E_p = E_{pol} - E_{non\text{-}pol}$ where E_{pol} is the total energy of the electron-doped V_2O_5 system where the excess electron localizes at one V center and $E_{non\text{-}pol}$ is the total energy of the non-polaronic system in which the injected electron spreads over entire V centers in the supercell. The non-polaronic state was obtained by adding an electron into the supercell without making any perturbations to the lattice. The symmetry imposed in the system forbids geometrical changes to accommodate the added electron at one V center leading to the partial occupation of all V centers in the supercell, as shown in Fig S2. The calculated E_p of –0.40 eV at U = 4.0 eV indicates that the excess electron prefers to localize at one V center and form a small polaron.

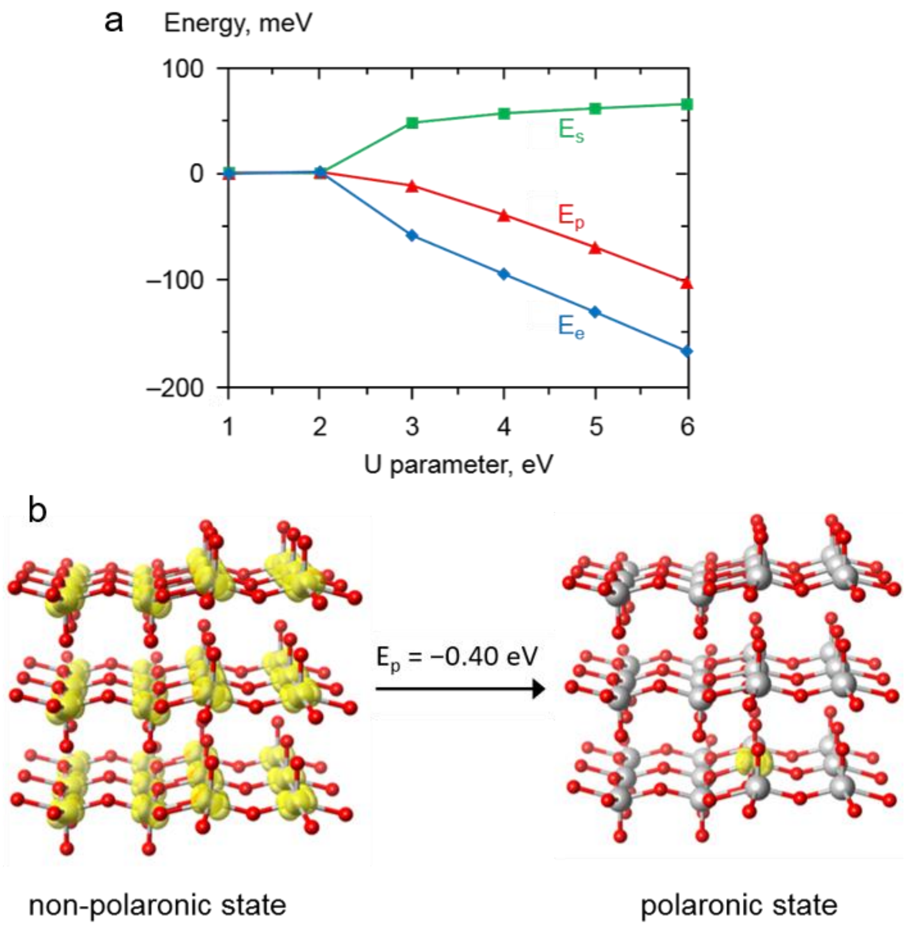

Fig. S2 a) Calculated polaron formation (E_p), lattice strain (E_s), and gained electronic energies (E_e) of the electron-doped V_2O_5 system at various U values. b) Schematic illustration of small polaron formation in the V_2O_5 lattice

Section S3. Position of the Li^+ ion in the V_2O_5 lattice

We present the most stable intercalated position of Li in the 1×3×3 V_2O_5 supercell. When a Li atom is introduced into the bulk V_2O_5, the Li atom ionizes to form a Li^+ ion and donates an extra electron to the V_2O_5 lattice. The extra electron localizes at the V center closest to the ionized Li^+ ion leading to a formation of a small polaron at the reduced V center. The most stable intercalated site of the Li^+ is at the hollow site above a ring formed by four units of VO_5 square-pyramids between V_2O_5 layers, Fig. S3a.

The position of Li^+ ion is off centro-symmetry due to the Coulombic attraction between positively charged Li^+ ion and negatively charged polaron and oxide anion, Fig. S3b. The deviated distance from the center was calculated to be 51 pm in which the point at the center was determined from the projected coordinates (x, y) of the four V centers. The distance between the Li^+ ion and the nearest V centers are $d_{Li\text{-}V1}$ = 311 pm, $d_{Li\text{-}V2a}$ = 313 pm, and $d_{Li\text{–}V2b}$ = 382 pm, as shown in Fig S1b. It can be seen that asymmetric deviation is more pronounce in the [010] direction than that of the [100] direction as the $d_{Li\text{-}V2by}$ is considerably larger than the $d_{Li\text{-}V1}$ and $d_{Li\text{-}V2a}$.

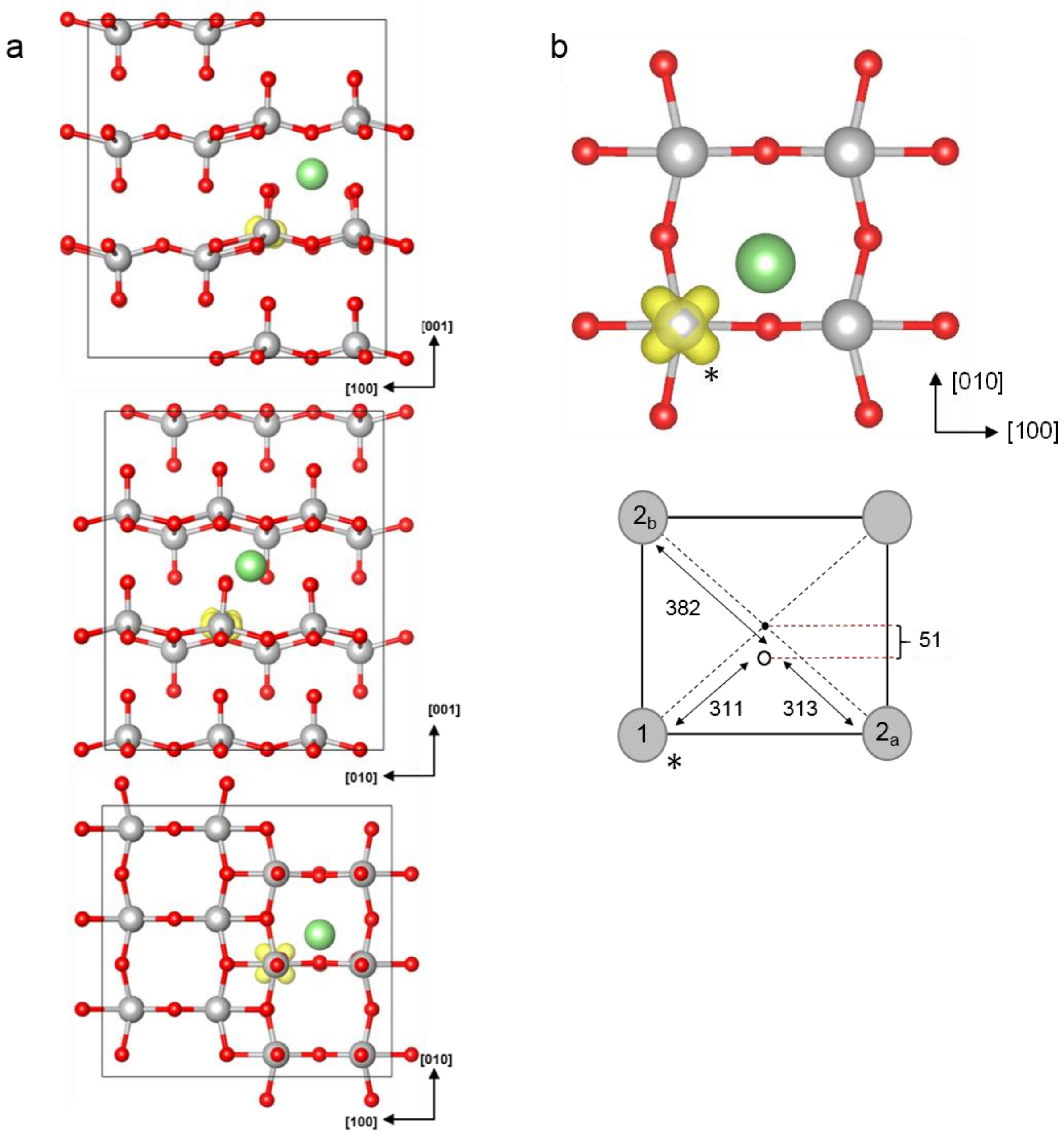

Fig. S3 a) Schematic illustration of most stable Li position in the V_2O_5 structure. b) Illustration of relative position of Li-ion in the four-membered VO_5 ring. The labelled numbers indicate the deviated distance from the center and distances between the Li^+ ion and the nearest V centers along [100] and [010] directions (both are in pm).

Section S4. Polaron hopping barriers as a function of U value

In order to understand the effect of U parameter on the polaron transport properties, we calculated the polaron migration barriers at various U values. The considered migration path is along the [010] direction in the pure V_2O_5 system. It can be seen from Fig. S4 that the calculated hopping barrier increases as the value of U becomes larger. For U values in the range of 3 to 7 eV, the migration barrier fluctuates within 0.17 and 0.29 eV. An extremely large barrier was found as the U value increased to 8 eV suggesting an over amplification of electron localization. Regarding the computations and previous computational studies,[1,2] the used U values in the range of 3 to 4 eV yield reasonable agreement of the calculated hopping barriers to that of the obtained experimental activation energy (0.23–0.28 eV).[3–5]

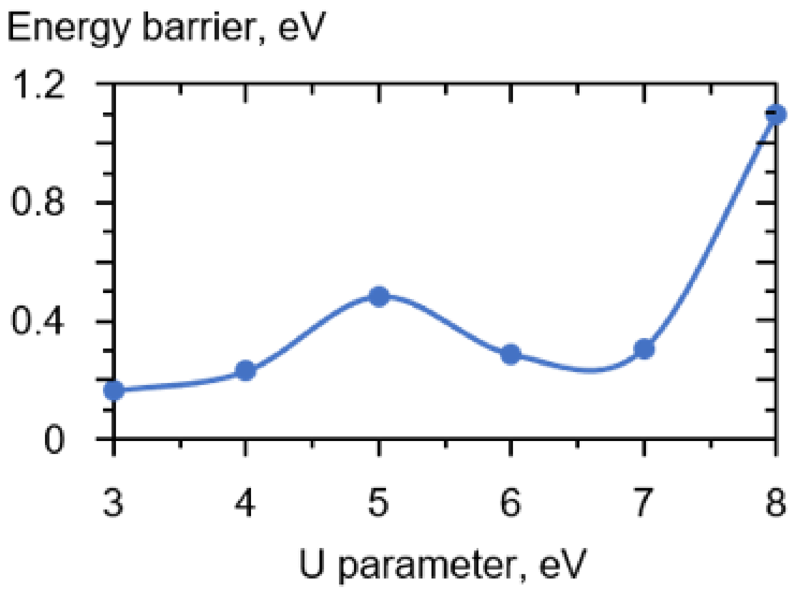

Fig. S4 Calculated polaron migration barriers along the [010] direction in the V_2O_5 lattice at various values of U parameter.

Section S5. Transition state structures obtained from LE and NEB calculations

The NEB-calculated TS configuration of the [010] hopping exhibits higher energy than that obtained from the LE computation. This is due to a relatively more strained TS structure obtained from the NEB calculation. As shown in Fig. S5a, the NEB-calculated TS structure involves shorter V–O3 bonds (191 pm) and smaller V*–O–V* bond angles (103°) as compared to the LE-calculated TS structure (193 pm and 104°). Such a structure with shorter bonds is not preferred to accommodate a small polaron resulting in a relatively high energy.

On the other hand, a more relaxed TS structure is achieved when using the NEB optimization for the [001] hopping. The NEB-calculated TS structure exhibits longer V*–O1 bond (164 pm) than that of the LE calculations (163 pm). In addition, the longer V*–O1 bond leads to a shorter O–V* distance across the van der Waals gap where the NEB calculations exhibit a somewhat smaller O–V* distance (269 vs 272 pm) which could facilitate polaron migration and lower the hopping barrier. As a result, the NEB-calculated TS configuration exhibits lower energy than that of the LE computation.

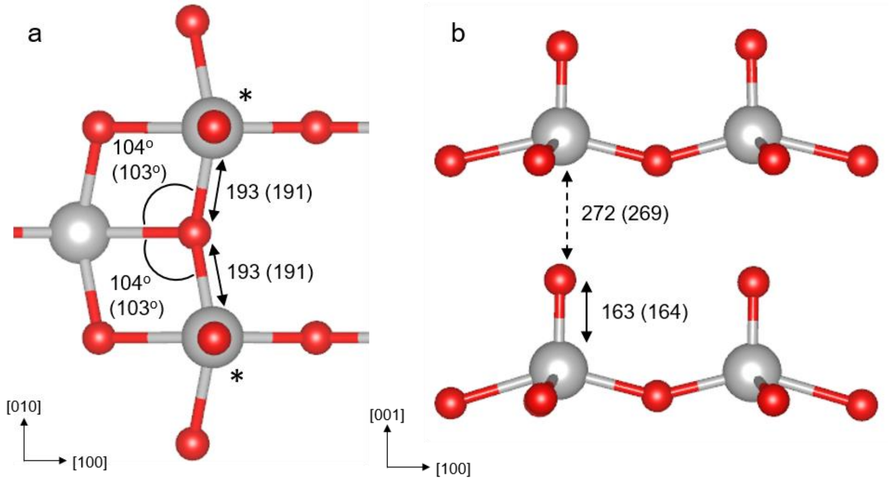

Fig. S5 Calculated TS structures of polaron migration along the a) [010] and b) [001] directions. The labelled numbers indicate the calculated bond lengths (pm) and bond angles obtained using the LE method and the NEB method (numbers in parentheses).

Section S6. Estimation of electronic coupling constant

In this study, we used the DFT+U method to calculate the adiabatic activation barriers of polaron hopping where the validity of Born-Oppenheimer approximation is applied. The adiabaticity of a polaron transfer can be determined if we know the adiabatic barrier and the electronic coupling constant (V_{AB}). We employed the method described in the computational work by Adelstein and co-workers to estimate the V_{AB}. V_{AB} is a half of the energy difference between the adiabatic bonding and antibonding electronic states at the TS, as follow

$$V_{AB} = \frac{1}{2} \Delta E_{12}$$

In practice, we can estimate the ΔE_{12} from the positions of the two gap states in the calculated density of states of the TS. The bonding state (below the Fermi energy) and the antibonding state (above the Fermi energy) must be in the gap between the valence band and the conduction band and should be linear combinations of the initial and final polaron states.

The calculated DOS of the TSs along all considered migration paths in the presence and absence of the Li^+ ion exhibit a single gap state indicating that the estimated ΔE_{12} and V_{AB} is negligible, as shown in Fig. S6. As a result, these polaron migration processes are non-adiabatic. Our prediction is consistent with the previous study that the polaron transport in the Li-intercalated V_2O_5 along the [010] direction is merely diabatic.[4] Their calculated V_{AB} obtained using the simplified fragment charge difference method[5] is very small, V_{AB} = 0.001 eV.

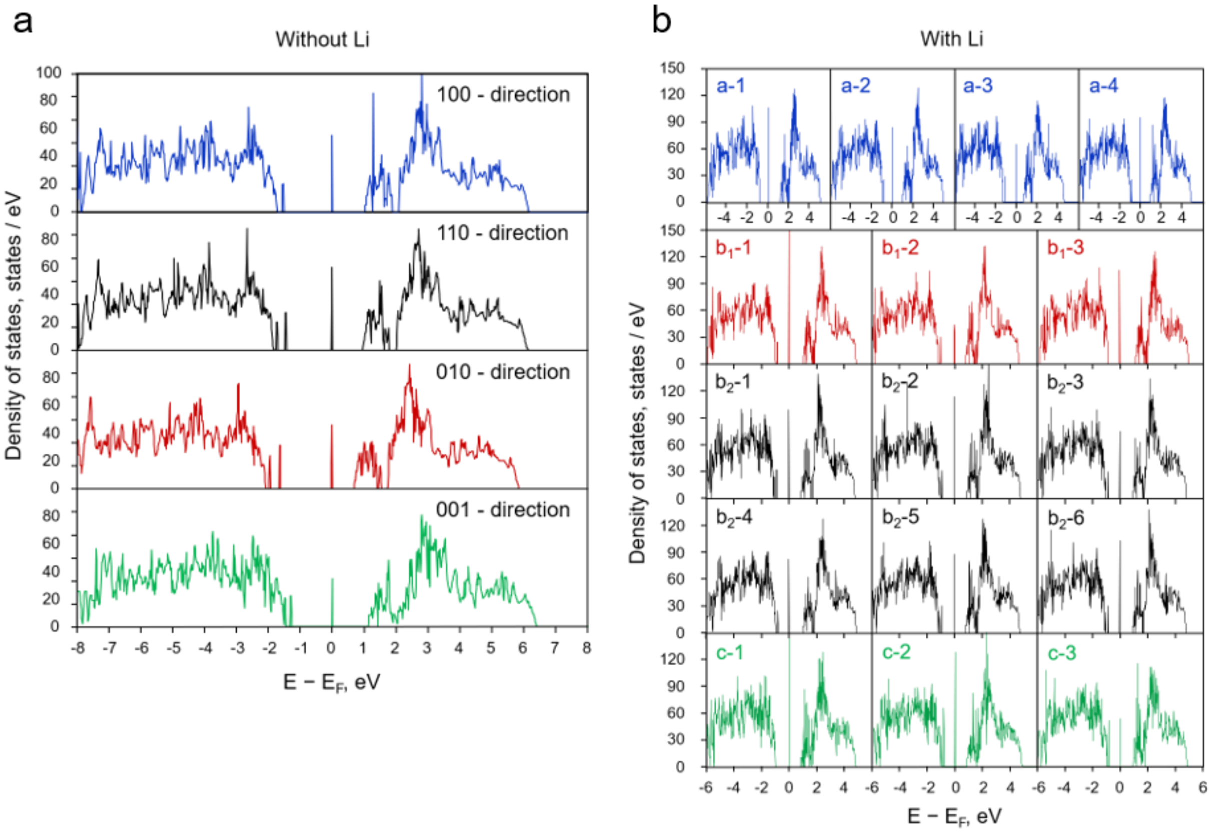

Fig. S6 Projected density of states of transition state configurations for all considered polaron migration paths a) in the absence of the Li^+ ion and b) in the presence of the Li^+ ion.

References

1. D. O. Scanlon, A. Walsh, B. J. Morgan and G. W. Watson, *J. Phys. Chem. C*, 2008, **112**, 9903–9911.
2. J. Carrasco, *J. Phys. Chem. C*, 2014, **118**, 19599–19607.
3. R. Enjalbert and J. Galy, *Acta Crystallogr. Sect. C: Cryst. Struct. Commun.*, 1986, **42**, 1467–1469.
4. S. Suthirakun, A. Genest and N. Rosch, *J. Phys. Chem. C*, 2018, **122**, 150–157.
5. A. A. Voityuk and N. Rösch, *The Journal of Chemical Physics*, 2002, **117**, 5607-5616.
